# Evaluation of the sarcopenia quality of life (SarQoL) questionnaire in community dwelling outpatient postmenopausal hungarian women

**DOI:** 10.1186/s12891-023-06454-2

**Published:** 2023-04-27

**Authors:** Zoltán Pap, Irina Kalabiska, Ádám Balogh, Harjit Pal Bhattoa

**Affiliations:** 1 Department of Traumatology and Hand Surgery, Faculty of Medicine, Kalman Laki Doctoral School of the University of Debrecen, University of Debrecen, Debrecen, Hungary; 2 Research Center for Sport Physiology, Hungarian University of Sports Science, Budapest, Hungary; 3 Regional Osteoporosis Center, Department of Obstetrics and Gynecology, Faculty of Medicine, University of Debrecen, Debrecen, Hungary; 4 Department of Laboratory Medicine, Faculty of Medicine, University of Debrecen, Nagyerdei Blvd. 98, Debrecen, H- 4032 Hungary

**Keywords:** Sarcopenia, Sarcopenia Quality of Life (SarQoL) questionnaire, Hungarian women

## Abstract

**Background:**

Sarcopenia is defined as an age-related progressive and systemic loss of muscle mass and function. World Health Organization (WHO) definition of health-related quality of life (QoL) states that health is considered “a state of complete physical, mental and social well-being and not merely the absence of disease or infirmity”, and a decline in QoL is anticipated in individuals with sarcopenia. Beaudart et al. framed the concept of defining QoL in patients suffering from sarcopenia (SarQoL) based on fundamental procedures of QoL questionnaire development, expert recommendations and studies. The aim of the present study is to evaluate the discriminative power, internal consistency and floor and ceiling effects using data available from a sarcopenia study published recently, where the Hungarian version of the SarQoL questionnaire was also administered.

**Methods:**

In this cross-sectional study, data from SarQoL questionnaire administered to a postmenopausal sarcopenia study cohort (n = 100) was scrutinized for evaluation of psychometric properties of the questionnaire. Our verification of the psychometric properties consisted of discriminative power analysis, assessment of internal consistency, and floor and ceiling effects. The homogeneity of the SarQoL questionnaire, i.e., its internal consistency was measured using Cronbach’s alpha coefficient. Correlation between the overall and domain SarQoL questionnaire scores and appendicular skeletal muscle mass in sarcopenic individuals was assessed. Furthermore, the difference of SarQoL overall and domain scores between sarcopenic and non-sarcopenic patients was also evaluated.

**Results:**

The median (interquartile range (IQR)) overall SarQoL questionnaire score was 81.5 (67.1–91.5). There was a statistically significant lower overall SarQoL score comparing sarcopenic and non-sarcopenic subjects median (IQR): 75.3 (62.1–86.3) vs. 83.7 (71.4–92.1); p = 0.041). The sarcopenic subjects showed a statistically significant (p = 0.021) correlation between the overall SarQoL score and appendicular skeletal muscle mass (Spearman’s ϱ = 0.412). The overall Cronbach’s alpha of 0.937 indicated a high internal consistency of the Hungarian version of the SarQoL questionnaire. No floor or ceiling effects were noted in the overall SarQoL questionnaire score.

**Conclusion:**

In our study on community dwelling outpatient postmenopausal Hungarian women, the overall score of the Hungarian version of the SarQoL questionnaire had significant discriminative power to distinguish between sarcopenic and non-sarcopenic patients, had high internal consistency and no floor or ceiling effects.

**Supplementary Information:**

The online version contains supplementary material available at 10.1186/s12891-023-06454-2.

## Background

Sarcopenia is defined as an age-related progressive and systemic loss of muscle mass and function [1]. This debilitating geriatric condition is being increasingly recognized globally as a considerable public health burden, where consequences of sarcopenia include impaired physical function and mobility, paralleled with augmented risk of falls, hospitalization and mortality [3–6].

Historically, mortality has been the prime indicator of public health. Modern medical innovations have substantially increased life expectancy, furthering the focus of medicine on quality of life (QoL) and hence a need for its objective evaluation [7, 8]. Subsequently, the term QoL has found increased dominance in healthcare [9]. As a consequence, construction and testing of instruments destined to measure health related QoL have been in focus [10–12]. The perception of health has undergone intense refinement, where a change in conception has encouraged assessment of dominantly positive endpoints rather than the traditionally favoured negative health outcomes [13].

World Health Organization (WHO) definition of health-related QoL states that health is considered “a state of complete physical, mental and social well-being and not merely the absence of disease or infirmity”, and a decline in QoL is anticipated in individuals with sarcopenia [14]. Until the endeavour by Beaudart et al. in 2015, QoL in sarcopenic subjects was judged merely by generic questionnaires which may not evidently be able to capture the indistinct effects of the condition [15]. Beaudart et al. framed the concept of defining QoL in patients suffering from sarcopenia (SarQoL) based on fundamental procedures of QoL questionnaire development, expert recommendations and studies [16–23]. The SarQoL was developed in French and validated by Beaudart et al. [24, 25]. Till date, the questionnaire has been translated to 30 languages and made available on the internet at www.sarqol.org. Furthermore, the English, Romanian, Dutch, Polish, Hungarian, Lithuanian, Russian, Greek, Ukranian, Serbian, Spanish, Korean, Chinese, and Turkish versions have been validated and its psychometric properties evaluated [26–41].

The Hungarian translation of the original SarQoL questionnaire was done by Hodinka et al. in 2018, and later validated by Greenick et al. in 2022 [30, 40]. The aim of the present study is to evaluate the discriminative power, internal consistency, and floor and ceiling effects using data available from a sarcopenia study published recently, where the Hungarian version of the SarQoL questionnaire was also administered [3].

## Methods

### Study population

Data from SarQoL questionnaire administered to a sarcopenia study cohort was scrutinized for evaluation of psychometric properties of the questionnaire [3]. In short, the cohort (n = 100) was studied at a center where post-menopausal women arriving for routine bone densitometry volunteered to participate following thorough briefing on the study concept and study procedures [3]. The sentinel findings of the study have been previously published and all study proceedures were performed adhering to the Declaration of Helsinki and an ethics approval was formally received from the competent local bodies (Approval no. 5314 − 2019) [3].

### The SarQoL

The SarQoL is constituted by 22 questions composed in total by 55 individual items that are rated on a 4-point Likert scale. The questionnaire is designed to give a maximum score of 100 points, where higher scores reflect better quality of life. The 55 items are sorted into seven individual domains, from domain 1 to domain 7. Individual domains address distinct features as follows: domain 1 - physical and mental health; domain 2 - locomotion; domain 3 - body composition; domain 4 - functionality; domain 5 - activities of daily living; domain 6 - leisure activities; domain 7 - fears. It is a self-administrated questionnaire that was designed to be completed in 10 min [15]. Complimentary personalized access is available upon registration and the overall and individual domain related scores are calculated upon entering the responses into the dedicated boxes on the online platform [42]. All completed questionnaires and calculated scores are stored and retrievable as desired. The Hungarian version of the questionnaire was administered in our cohort [30].

Our verification of the psychometric properties consisted of discriminative power analysis, assessment of internal consistency, and floor and ceiling effects. As suggested by Beaudart et al., discriminative power analysis was executed on the whole study population (n = 100) and the latter two analyses were done in those where the diagnosis of sarcopenia was confirmed as per the EWGSOP2 definition (n = 31) [26, 43].

To analyse the discriminative power of the questionnaire, it is assumed that SarQoL score is higher in those without sarcopenia as compared to sarcopenic subjects [26]. Our study population confirmed to the EWGSOP2 definition for sarcopenia, with low muscle strength and low muscle quantity [43]. Muscle strength was assessed with a handgrip dynamometer and appendicular skeletal muscle mass by dual-energy X-ray absorptiometry whole body scan using a LUNAR Prodigy (GE-Lunar Corp., Madison, WI, USA) densitometer [3].

Correlation analyses was performed between the overall and domain SarQoL questionnaire scores and appendicular skeletal muscle mass in sarcopenic individuals. The homogeneity of the SarQoL questionnaire, i.e., its internal consistency was measured using Cronbach’s alpha coefficient [25].

Floor and ceiling effects for the overall and individual domain SarQoL scores were noted when the lowest or the highest score were achieved by the subject. Floor and ceiling effects higher than 15% among the scores by the subjects were considered significant [44].

### Statistical analysis

The normality of distribution was assessed using the Kolmogorov-Smirnov test. The Spearman’s ϱ was calculated for correlation analysis. A Spearman’s ϱ value above 0.81, between 0.61 and 0.80, between 0.41 and 0.60, between 0.21 and 0.40, and less than 0.20 were evaluated as excellent, very good, good, acceptable and insufficient, respectively [45]. The Mann-Whitney U test was performed to assess the difference of SarQoL overall and domain scores between sarcopenic and non-sarcopenic patients. Odds ratio (95% CI) was used to measure the relationship between overall and individual domains of the SarQoL questionnaire scores and the likelihood of sarcopenia, using binary logistic regression. Cronbach’s alpha coefficient was calculated to assess internal consistency of the SarQoL questionnaire. A Cronbach’s alpha coefficient value of greater than 0.70 was considered as a high level of internal consistency [46]. Statistically significant difference was defined as p<0.05. The SPSS Statistics software, version 29.0 (IBM Corps., Armonk, NY, USA) was used to perform all statistical analyses.

## Results

All participants (n = 100) in the study completed the SarQoL questionnaire. The median (interquartile range (IQR)) overall and individual domain SarQoL questionnaire scores calculated by entering the responses of all questions in the questionnaires into SarQoL website are presented in Table 1. There was a statistically significant lower overall SarQoL score comparing sarcopenic and non-sarcopenic subjects (median (IQR): 75.3 (62.1–86.3) vs. 83.7 (71.4–92.1); p = 0.041). Among the individual domains, D2 locomotion (72.2 (55.6–88.9) vs. 86.1 (69.4–97.2); p = 0.008) and D5 activities of daily living (78.3 (55.0-88.3) vs. 88.3 (75.8–94.1); p = 0.012) were the only 2 domains out of the total 7 domains that showed a statistically significant difference between the sarcopenic and the non-sarcopenic individuals. Additionally, the likelihood of sarcopenia was statistically significantly predicted by the overall SarQoL questionnaire score, D2 locomotion domain SarQoL questionnaire score and D5 activities of daily living domain SarQoL questionnaire score with odds ratios (95%CI) of 0.967 (0.942–0.997), 0.970 (0.948–0.993) and 0.965 (0.940–0.990), respectively (Table 2). The sarcopenic subjects showed a statistically significant correlation between the overall SarQoL score (Spearman’s ϱ = 0.412), domain 2 – locomotion SarQoL questionnaire score (Spearman’s ϱ =0.372), domain 3 – body composition SarQoL questionnaire score (Spearman’s ϱ =0.439) and domain 5 activities of daily living SarQoL questionnaire score (Spearman’s ϱ = 0.372) and appendicular skeletal muscle mass (Table 3).

**Table 1:** Results of the SarQoL questionnaire for sarcopenic and non-sarcopenic subjects

Variable	All participants	Sarcopenic (ASM < 15 kg)	Non-sarcopenic (ASM ≥ 15 kg)	*p* value
	(n = 100)	(n = 31)	(n = 69)	
SarQoL Overall	81.5 (67.1–91.5)	75.3 (62.1–86.3)	83.7 (71.4–92.1)	0.041
SarQoL D1 Physical and mental health	80.0 (65.5–91.6)	78.9 (58.9–90.0)	83.3 (68.9–92.2)	0.183
SarQoL D2 Locomotion	86.1 (63.9–94.4)	72.2 (55.6–88.9)	86.1 (69.4–97.2)	0.008
SarQoL D3 Body composition	83.3 (70.8–98.3)	79.2 (62.5–91.7)	83.3 (70.8–100)	0.336
SarQoL D4 Functionality	80.8 (62.7–90.4)	78.8 (60.7–86.5)	82.7 (68.2–90.4)	0.275
SarQoL D5 Activities of daily living	85.0 (68.3–91.7)	78.3 (55.0-88.3)	88.3 (75.8–94.1)	0.012
SarQoL D6 Leisure activities	66.5 (49.9–66.5)	66.5 (49.9–66.5)	66.5 (49.9–66.5)	0.713
SarQoL D7 Fears	87.5 (75–100)	75 (75–100)	100 (75–100)	0.100

Median (interquartile range); ASM: Appendicular skeletal muscle mass; SarQoL: Sarcopenia quality of life

**Table 2:** Discriminitive power of the SarQoL questionnaire (n = 100)

Variable	Odds ratio (95% CI)	*p* value
SarQoL Overall	0.967 (0.942–0.997)	0.029
SarQoL D1 Physical and mental health	0.977 (0.952–1.003)	0.083
SarQoL D2 Locomotion	0.970 (0.948–0.993)	0.011
SarQoL D3 Body composition	0.984 (0.959–1.010)	0.215
SarQoL D4 Functionality	0.984 (0.959–1.010)	0.225
SarQoL D5 Activities of daily living	0.965 (0.940–0.990)	0.007
SarQoL D6 Leisure activities	0.995 (0.967–1.023)	0.716
SarQoL D7 Fears	0.977 (0.950–1.006)	0.117

**Table 3:** Correlation between SarQoL questionnaire scores and appendicular skeletal muscle mass in the sarcopenia population (n = 31)

Variable	Spearman’s ϱ	*p* value
SarQoL overall	0.412	0.021
SarQoL D1 Physical and mental health	0.304	0.097
SarQoL D2 Locomotion	0.372	0.039
SarQoL D3 Body composition	0.439	0.014
SarQoL D4 Functionality	0.351	0.053
SarQoL D5 Activities of daily living	0.372	0.039
SarQoL D6 Leisure activities	0.028	0.883
SarQoL D7 Fears	0.292	0.111

The overall Cronbach’s alpha of 0.937 indicated a high internal consistency of the Hungarian version of the SarQoL questionnaire. The Cronbach’s alpha varied between 0.917, when deleting domain 4 – functionality, and 0.945, when deleting domain 6 – leisure activities. Additionally, all domain scores showed a statistically significant correlation with the overall score varying between a Spearman’s ϱ value of 0.529, for domain 6 – leisure activities, and 0.949 for domain 5 – activities of daily living (Table 4).

**Table 4:** Internal Consistency and Spearmans’ correlation analyses in the sarcopenia population (n = 31)

Variable	Correlations between overall and domain scores		Cronbach’s α if domain deleted	Overall Cronbach’s α
	Spearman’s ϱ	*p* value		
SarQoL D1 Physical and mental health	0.849	< 0.001	0.926	0.937
SarQoL D2 Locomotion	0.877	< 0.001	0.927	
SarQoL D3 Body composition	0.853	< 0.001	0.924	
SarQoL D4 Functionality	0.934	< 0.001	0.917	
SarQoL D5 Activities of daily living	0.949	< 0.001	0.921	
SarQoL D6 Leisure activities	0.529	0.002	0.945	
SarQoL D7 Fears	0.815	< 0.001	0.930	

No sarcopenic subject presented either the lowest or the highest overall SarQoL questionnaire score. Consequently, there was neither floor nor ceiling effects. However, upon analysing the floor and ceiling effects at the individual domain level, domain 3 - body composition and domain 7 - fears had a significant ceiling effect of 22.6% and 32,3%, respectively. Non-significant (<15%) ceiling effects were noted for domain 1 – physical and mental health (9.7%), domain 2 – locomotion (3.2%) and domain 5 – activities of daily living (3.2%). No ceiling effect was noted for domain 4 – functionality and domain 6 – leisure activities. Floor effects were not noticed for any of the individual domains.

### Discussion

Inaugurated to the scientific community in 2015, Beaudart et al. developed and validated the first quality of life questionnaire specific for sarcopenia [24, 25]. Table 5 summarizes the results of studies that have validated the SarQoL questionnaire translated into different languages. It is evident that cohorts of various sizes with variable percentage of subjects with sarcopenia have been studied. On the same note, it can also be recognized that only 7 of these studies were able to align with the requirement that demands inclusion of at least 50 subjects with sarcopenia to evaluate internal consistency and floor and ceiling effects of the questionnaire [29, 31–33, 36–38, 44].

**Table 5:** Comparison of SarQoL questionnaire validation studies

Study	Cohort size	Patients with Sarcopenia	Internal consistency (Overall Cronbach’s α)	Discrimitation power* (*p* value)
Beaudart et al. (French) [24]	296	43	0.870	< 0.001
Beaudart et al. (English) [25]	297	14	0.880	0.01
Gasparik et al. (Romanian) [26]	100	13	0.946	0.018
Geerinck et al. (Dutch) [27]	92	30	0.883	0.003
Konstantynowicz et al. (Polish) [28]	106	60	0.920	0.013
Alekna et al. (Lithuanian) [30]	176	58	0.950	< 0.001
Safonova YA et al. (Russian) [31]	100	50	0.924	< 0.001
Tsekoura et al. (Greek) [32]	176	50	0.960	< 0.001
Dzhus et al. (Ukranian) [33]	49	28	0.898	0.014
Matijevic R et al. (Serbian) [34]	699	12	0.870	0.155
Fabrega-Cuadros R et al. (Spanish) [35]	252	66	0.904	0.008
Yoo JI et al. (Korean) [36]	450	53	0.866	< 0.001
Le X et al. (Chinese) [37]	159	51	0.867	< 0.001
Erdogan T et al. (Turkish) [38]	100	27	0.880	< 0.001
Montero-Errasquin B et al. (Spanish) [40]	86	16	0.840	0.008
Present study (Hungarian)	100	31	0.937	0.041

*Total SarQoL questionnaire scores: sarcopenia vs. non-sarcopenia

Furthermore, there is a lack in uniform application of the definition of sarcopenia among the various studies, we used the EWGSOP2 definition and used dual energy x-ray absorptiometry to determine lean muscle mass [3]. It perhaps needs to be emphasized that at the time of the conception of the SarQoL questionnaire by Beaudart et al., the EWGSOP definition was in use [24, 47]. The EWGSOP2 definition for sarcopenia was published in 2019, all SarQoL studies executed thereafter chose to use this updated definition to define sarcopenia in their study population with the exception of Le at al who used the AWGS 2019 definition [34, 39, 41, 43, 48].

Recruitment of low number of subjects generally deters statistical power, typically a power analysis is demanded but since the prevalence of sarcopenia is currently being mapped in various populations approaches to define the number of individuals to be included for a robust statistical analyses may be hindered. A limitation of our study includes the drawback that data from only 31 sarcopenic patients was available to evaluate the internal consistency and floor and ceiling effects of the SarQoL questionnaire instead of the recommended 50 subjects [44].

Depletion in estrogen levels particularly during menopause may cause decline in lean body mass [49, 50]. As such, perhaps inclusion of both sexes in sarcopenia studies may dilute the interpretation regarding various research questions, additionally, cohorts where both sexes have been included to draw inference may inherently inhibit plausible exploration of the research hypothesis [51]. Furthermore, heterogeneity of the study protocols of the various studies published on the topic inhibit head to head comparison.

Although the discriminative power of the overall SarQoL questionnaire score has been validated by most studies, the results obtained for individual domains have been heterogenous. During the validation of the original French SarQoL questionnaire and later the Dutch, Lithuanian, Russian, Greek and Turkish versions, scores for all the individual 7 domains were significantly lower in the sarcopenic patients as compared to their non-sarcopenic counterparts [25, 28, 31, 33, 39]. In other validation studies, for the English version D3, D6, and D7; for the Romanian version D4 and D6; for the Polish version D4, D6 and D7; for the Ukrainian version D2 and D6; for the Spanish study by Fabrega-Cuadros et al. D2, for the Chinese version D6; and for the Spanish study by Montero-Errasquin et al. D2, D3, D6 and D7 domain scores were not significantly lower in subjects with sarcopenia [26, 27, 29, 34, 36, 38, 41]. The study by Matijevic et al., using the Serbian version, found no statistically significant difference in the overall and individual domain score comparing the sarcopenic to the non-sarcopenic study participants [35]. The most probable reason behind this unsignificant difference is that they compared 687 non-sarcopenic subjects to only 12 sarcopenic subjects [35]. In our study, the overall SarQoL questionnaire score was statistically significantly lower in the sarcopenic as compared to the non-sarcopenic subjects, nonetheless, among the individual domains, D1, D3, D4, D6 and D7 were not significantly lower in the sarcopenic subject. Although various studies found nonsignificant lower scores in sarcopenic subjects in various individual domains, domain D6 – leisure activities is the common denominator in all. The reason here may perhaps, as proposed previously by Konstantynowicz et al., be the cultural difference particularly in leisure activities and a lack of robust sample size of the cohorts studied [29].

The internal consistency of the Hungarian version of the SarQoL questionnaire administered in our cohort was of high level and there was a statistically significant correlation between the overall and individual domain scores. This is in tally with all the previously published validation studies [25–29, 31–41].

Significant ceiling effects were noted in the individual domain D3 and D7 in our study cohort. Previously, significant ceiling effect was reported by Dzhus et al. in their Ukrainian cohort for domain D7 [51]. A plausible explanation may pertain to cross-cultural sensitivity of the questions in domain D3 and D7, nonetheless, this notion is rebutted by Greenick et al., where they validated the Hungarian version of the SarQoL in Romanian subjects with Hungarian mother tongue and found no floor or ceiling effects [40]. Although the cohort recruited for this validation study had no sarcopenic subjects [40].

To the best of our knowledge, test-retest reliability of the Hungarian version of the SarQoL questionnaire is still pending [40].

Given the ever increasing body of knowledge pertaining to the SarQoL questionnaire, it may well be envisaged that changes in its score could perhaps help evaluate efficiency in future interventional studies. Nonetheless, increased awareness of sarcopenia and the ever increasing volume of data exclamating the health economics of the condition may propel better efforts into the identification and diagnosis of the condition, furthermore, SarQoL may very well provide an objective approach to relinquish the gap in better understanding the daily impact of the condition on the quality of life of the patients.

## Conclusion

In conclusion, the overall score of the Hungarian version of the SarQoL questionnaire has significant discriminative power to distinguish between sarcopenic and non-sarcopenic patients, has high internal consistency and no floor or ceiling effects.

## Electronic supplementary material

Below is the link to the electronic supplementary material.

Supplementary Material 1

## Data Availability

All data generated or analyzed during this study are included in this published article and its supplementary information file.
