# Supplementary material for: Evaluation of the sarcopenia quality of life (SarQoL) questionnaire in community dwelling outpatient postmenopausal hungarian women
Source: BMC Musculoskelet Disord. 2023 Apr 27;24:331. doi: 10.1186/s12891-023-06454-2 (PMC10134597; doi:10.1186/s12891-023-06454-2)
Supplement: Supplementary file 1 — Supplementary Material 1 [file 12891_2023_6454_MOESM1_ESM.docx]

Supplementary data. All data generated or analyzed during the study.

| ID | DOE | ASM_kg | SarQoL | D1 | D2 | D3 | D4 | D5 | D6 | D7 |
| --- | --- | --- | --- | --- | --- | --- | --- | --- | --- | --- |
| 1 | 09.Jan.19 | 15.57 | 78.6 | 76.6 | 88.9 | 75.0 | 92.3 | 63.3 | 49.9 | 100.0 |
| 2 | 09.Jan.19 | 15.31 | 58.1 | 65.5 | 63.9 | 66.7 | 59.6 | 48.1 | 16.6 | 87.5 |
| 3 | 10.Jan.19 | 17.97 | 86.6 | 83.3 | 88.9 | 83.3 | 86.5 | 88.3 | 66.5 | 100.0 |
| 4 | 10.Jan.19 | 13.39 | 49.9 | 45.5 | 52.8 | 54.2 | 57.7 | 40.0 | 33.3 | 87.5 |
| 5 | 10.Jan.19 | 17.51 | 90.9 | 83.3 | 100.0 | 100.0 | 90.4 | 96.7 | 16.6 | 100.0 |
| 6 | 11.Jan.19 | 13.27 | 87.8 | 90.0 | 88.9 | 91.7 | 81.3 | 91.7 | 66.5 | 100.0 |
| 7 | 11.Jan.19 | 14.49 | 43.1 | 42.3 | 50.0 | 58.3 | 37.5 | 33.3 | 33.3 | 62.5 |
| 8 | 14.Jan.19 | 13.29 | 90.4 | 92.3 | 86.1 | 75.0 | 89.3 | 98.3 | 66.5 | 100.0 |
| 9 | 14.Jan.19 | 18.38 | 65.0 | 70.0 | 55.6 | 79.2 | 63.5 | 65.0 | 66.5 | 75.0 |
| 10 | 15.Jan.19 | 13.38 | 88.2 | 90.0 | 80.6 | 100.0 | 91.1 | 88.3 | 66.5 | 100.0 |
| 11 | 18.Jan.19 | 16.27 | 88.8 | 83.3 | 91.7 | 75.0 | 94.2 | 91.7 | 49.9 | 100.0 |
| 12 | 23.Jan.19 | 15.81 | 63.0 | 62.2 | 75.0 | 79.2 | 59.6 | 60.0 | 33.3 | 62.5 |
| 13 | 23.Jan.19 | 16.02 | 86.3 | 100.0 | 58.3 | 100.0 | 96.4 | 85.0 | 66.5 | 100.0 |
| 14 | 24.Jan.19 | 14.72 | 80.2 | 86.6 | 83.3 | 70.8 | 80.8 | 83.3 | 33.3 | 75.0 |
| 15 | 24.Jan.19 | 12.81 | 91.7 | 96.6 | 88.9 | 87.5 | 92.3 | 93.3 | 66.5 | 100.0 |
| 16 | 24.Jan.19 | 14.39 | 93.8 | 96.6 | 100.0 | 93.3 | 90.4 | 96.7 | 66.5 | 100.0 |
| 17 | 24.Jan.19 | 13.60 | 83.3 | 80.0 | 90.6 | 83.3 | 86.5 | 78.3 | 66.5 | 100.0 |
| 18 | 28.Jan.19 | 14.28 | 76.8 | 73.3 | 88.9 | 83.3 | 67.3 | 78.3 | 66.5 | 87.5 |
| 19 | 28.Jan.19 | 15.73 | 97.7 | 100.0 | 97.2 | 100.0 | 98.1 | 100.0 | 66.5 | 100.0 |
| 20 | 28.Jan.19 | 15.79 | 93.4 | 93.3 | 100.0 | 100.0 | 94.2 | 90.0 | 66.5 | 100.0 |
| 21 | 29.Jan.19 | 17.78 | 74.7 | 65.5 | 72.2 | 75.0 | 78.8 | 78.3 | 66.5 | 75.0 |
| 22 | 29.Jan.19 | 14.37 | 53.6 | 78.9 | 61.1 | 66.7 | 48.1 | 39.3 | 33.3 | 62.5 |
| 23 | 29.Jan.19 | 12.58 | 93.0 | 93.3 | 97.2 | 100.0 | 87.5 | 96.7 | 66.5 | 100.0 |
| 24 | 29.Jan.19 | 14.74 | 91.7 | 86.6 | 93.8 | 100.0 | 90.4 | 95.0 | 66.5 | 100.0 |
| 25 | 29.Jan.19 | 19.01 | 73.6 | 68.9 | 69.4 | 79.2 | 69.6 | 80.0 | 66.5 | 87.5 |
| 26 | 30.Jan.19 | 14.19 | 89.3 | 88.9 | 97.2 | 75.0 | 86.5 | 91.7 | 66.5 | 100.0 |
| 27 | 30.Jan.19 | 13.03 | 75.3 | 72.2 | 66.7 | 62.5 | 78.6 | 85.0 | 49.9 | 75.0 |
| 28 | 31.Jan.19 | 19.21 | 96.3 | 100.0 | 97.2 | 100.0 | 92.3 | 100.0 | 66.5 | 100.0 |
| 29 | 31.Jan.19 | 10.81 | 52.4 | 55.5 | 55.6 | 58.3 | 48.1 | 55.0 | 16.6 | 62.5 |
| 30 | 31.Jan.19 | 19.16 | 81.7 | 78.9 | 88.9 | 79.2 | 80.4 | 83.3 | 66.5 | 75.0 |
| 31 | 01.Feb.19 | 15.80 | 96.1 | 96.6 | 100.0 | 87.5 | 96.4 | 98.3 | 66.5 | 100.0 |
| 32 | 01.Feb.19 | 19.29 | 94.9 | 100.0 | 100.0 | 100.0 | 94.6 | 91.7 | 66.5 | 100.0 |
| 33 | 04.Feb.19 | 12.86 | 83.7 | 86.6 | 83.3 | 79.2 | 82.1 | 88.3 | 66.5 | 75.0 |
| 34 | 04.Feb.19 | 15.76 | 62.9 | 65.5 | 69.4 | 58.3 | 53.8 | 68.3 | 49.9 | 62.5 |
| 35 | 04.Feb.19 | 15.89 | 59.5 | 58.9 | 52.8 | 70.8 | 51.9 | 65.0 | 66.5 | 75.0 |
| 36 | 05.Feb.19 | 15.20 | 65.1 | 48.9 | 66.7 | 54.2 | 70.8 | 66.7 | 66.5 | 87.5 |
| 37 | 06.Feb.19 | 17.50 | 77.3 | 65.5 | 77.8 | 79.2 | 73.1 | 88.3 | 49.9 | 87.5 |
| 38 | 06.Feb.19 | 11.06 | 45.6 | 44.4 | 38.9 | 50.0 | 46.2 | 50.0 | 33.3 | 50.0 |
| 39 | 06.Feb.19 | 17.97 | 93.8 | 96.6 | 94.4 | 100.0 | 94.2 | 93.3 | 66.5 | 100.0 |
| 40 | 06.Feb.19 | 16.38 | 70.3 | 68.9 | 69.4 | 70.8 | 71.2 | 75.0 | 33.3 | 75.0 |
| 41 | 07.Feb.19 | 16.01 | 62.1 | 55.5 | 63.9 | 54.2 | 62.5 | 63.3 | 66.5 | 75.0 |
| 42 | 07.Feb.19 | 16.04 | 89.0 | 90.0 | 91.7 | 91.7 | 90.4 | 86.7 | 66.5 | 100.0 |
| 43 | 07.Feb.19 | 11.85 | 73.8 | 78.9 | 72.2 | 66.7 | 78.8 | 70.0 | 66.5 | 75.0 |
| 44 | 07.Feb.19 | 24.10 | 94.0 | 100.0 | 94.4 | 100.0 | 94.6 | 91.7 | 66.5 | 100.0 |
| 45 | 08.Feb.19 | 16.90 | 44.5 | 51.1 | 33.3 | 41.7 | 42.3 | 43.3 | 66.5 | 75.0 |
| 46 | 08.Feb.19 | 15.96 | 56.1 | 48.9 | 58.3 | 62.5 | 53.8 | 55.0 | 66.5 | 75.0 |
| 47 | 13.Feb.19 | 18.39 | 81.3 | 93.3 | 63.9 | 79.2 | 84.6 | 85.0 | 49.9 | 100.0 |
| 48 | 13.Feb.19 | 14.07 | 78.6 | 83.3 | 66.7 | 66.7 | 76.8 | 90.0 | 49.9 | 87.5 |
| 49 | 14.Feb.19 | 15.66 | 68.6 | 58.9 | 69.4 | 91.7 | 61.5 | 78.3 | 33.3 | 75.0 |
| 50 | 14.Feb.19 | 12.89 | 97.7 | 100.0 | 100.0 | 100.0 | 98.1 | 98.3 | 66.5 | 100.0 |
| 51 | 14.Feb.19 | 14.74 | 92.2 | 88.9 | 100.0 | 100.0 | 86.5 | 95.0 | 66.5 | 100.0 |
| 52 | 18.Feb.19 | 13.48 | 68.7 | 75.5 | 55.6 | 75.0 | 75.0 | 66.7 | 66.5 | 75.0 |
| 53 | 18.Feb.19 | 15.36 | 84.3 | 80.0 | 86.1 | 91.7 | 88.5 | 85.0 | 33.3 | 100.0 |
| 54 | 19.Feb.19 | 14.38 | 70.1 | 72.2 | 61.1 | 62.5 | 69.2 | 76.7 | 66.5 | 75.0 |
| 55 | 19.Feb.19 | 20.63 | 93.0 | 100.0 | 97.2 | 100.0 | 96.4 | 85.0 | 66.5 | 100.0 |
| 56 | 19.Feb.19 | 14.83 | 97.2 | 100.0 | 94.4 | 100.0 | 100.0 | 98.3 | 66.5 | 100.0 |
| 57 | 19.Feb.19 | 15.28 | 80.8 | 80.0 | 100.0 | 83.3 | 64.3 | 88.3 | 33.3 | 100.0 |
| 58 | 19.Feb.19 | 15.89 | 63.7 | 62.2 | 55.6 | 54.2 | 61.5 | 75.0 | 49.9 | 62.5 |
| 59 | 19.Feb.19 | 16.21 | 87.9 | 92.2 | 91.7 | 100.0 | 78.8 | 90.0 | 66.5 | 100.0 |
| 60 | 20.Feb.19 | 18.24 | 51.9 | 58.9 | 52.8 | 50.0 | 38.5 | 56.7 | 66.5 | 62.5 |
| 61 | 20.Feb.19 | 16.41 | 90.0 | 86.6 | 91.7 | 91.7 | 82.7 | 98.3 | 66.5 | 100.0 |
| 62 | 21.Feb.19 | 16.35 | 96.8 | 96.6 | 100.0 | 100.0 | 96.2 | 98.3 | 66.5 | 100.0 |
| 63 | 21.Feb.19 | 15.10 | 92.9 | 96.6 | 100.0 | 91.7 | 88.5 | 93.3 | 66.5 | 100.0 |
| 64 | 21.Feb.19 | 18.54 | 86.7 | 78.9 | 86.1 | 91.7 | 87.5 | 91.7 | 66.5 | 87.5 |
| 65 | 21.Feb.19 | 18.36 | 85.1 | 80.0 | 97.2 | 75.0 | 75.0 | 91.7 | 66.5 | 100.0 |
| 66 | 21.Feb.19 | 19.27 | 79.6 | 88.9 | 75.0 | 100.0 | 73.1 | 81.7 | 66.5 | 75.0 |
| 67 | 21.Feb.19 | 17.31 | 76.7 | 65.5 | 77.8 | 83.3 | 78.8 | 80.0 | 66.5 | 75.0 |
| 68 | 22.Feb.19 | 19.29 | 42.4 | 55.5 | 27.8 | 50.0 | 40.4 | 43.3 | 33.3 | 62.5 |
| 69 | 25.Feb.19 | 16.63 | 84.9 | 85.5 | 88.9 | 100.0 | 73.1 | 91.7 | 66.5 | 87.5 |
| 70 | 25.Feb.19 | 17.34 | 93.4 | 93.3 | 100.0 | 91.7 | 90.4 | 65.0 | 66.5 | 100.0 |
| 71 | 25.Feb.19 | 17.32 | 66.7 | 75.5 | 63.9 | 79.2 | 57.7 | 68.3 | 66.5 | 75.0 |
| 72 | 27.Feb.19 | 20.00 | 92.1 | 100.0 | 97.2 | 83.3 | 87.5 | 93.3 | 66.5 | 100.0 |
| 73 | 27.Feb.19 | 15.58 | 58.2 | 48.9 | 55.6 | 54.2 | 57.7 | 64.3 | 66.5 | 62.5 |
| 74 | 01.Mar.19 | 18.31 | 63.8 | 62.2 | 75.0 | 70.8 | 60.7 | 57.1 | 66.5 | 75.0 |
| 75 | 04.Mar.19 | 17.01 | 92.4 | 100.0 | 97.2 | 83.3 | 86.5 | 95.0 | 66.5 | 100.0 |
| 76 | 04.Mar.19 | 14.21 | 62.0 | 62.2 | 58.3 | 70.8 | 55.8 | 70.0 | 33.3 | 75.0 |
| 77 | 04.Mar.19 | 15.97 | 90.1 | 90.0 | 86.1 | 91.7 | 96.4 | 88.3 | 66.5 | 100.0 |
| 78 | 04.Mar.19 | 17.05 | 72.6 | 68.9 | 83.3 | 70.8 | 60.7 | 80.0 | 66.5 | 75.0 |
| 79 | 04.Mar.19 | 15.05 | 81.2 | 78.9 | 86.1 | 75.0 | 78.8 | 88.3 | 33.3 | 87.5 |
| 80 | 05.Mar.19 | 16.52 | 93.8 | 86.6 | 94.4 | 100.0 | 92.3 | 100.0 | 66.5 | 100.0 |
| 81 | 05.Mar.19 | 14.07 | 79.6 | 88.9 | 75.0 | 100.0 | 73.1 | 81.7 | 66.5 | 75.0 |
| 82 | 05.Mar.19 | 20.93 | 76.4 | 72.2 | 72.2 | 70.8 | 76.8 | 83.3 | 66.5 | 75.0 |
| 83 | 05.Mar.19 | 16.28 | 57.5 | 75.5 | 38.9 | 70.8 | 59.6 | 53.3 | 66.5 | 62.5 |
| 84 | 06.Mar.19 | 19.71 | 84.9 | 72.2 | 91.7 | 100.0 | 86.5 | 86.7 | 66.5 | 75.0 |
| 85 | 07.Mar.19 | 15.99 | 81.9 | 80.0 | 97.2 | 70.8 | 80.8 | 78.3 | 66.5 | 87.5 |
| 86 | 07.Mar.19 | 15.42 | 80.6 | 72.2 | 75.0 | 83.3 | 87.5 | 88.3 | 33.3 | 75.0 |
| 87 | 08.Mar.19 | 15.50 | 83.0 | 83.3 | 97.2 | 100.0 | 75.0 | 81.7 | 66.5 | 75.0 |
| 88 | 08.Mar.19 | 13.83 | 88.3 | 88.9 | 80.6 | 91.7 | 86.5 | 95.0 | 66.5 | 100.0 |
| 89 | 08.Mar.19 | 15.68 | 88.3 | 88.9 | 80.6 | 91.7 | 86.5 | 95.0 | 66.5 | 100.0 |
| 90 | 08.Mar.19 | 15.75 | 93.5 | 90.0 | 100.0 | 100.0 | 98.2 | 88.3 | 66.5 | 100.0 |
| 91 | 08.Mar.19 | 11.04 | 66.3 | 65.5 | 58.3 | 70.8 | 57.7 | 78.3 | 66.5 | 62.5 |
| 92 | 11.Mar.19 | 14.61 | 94.4 | 93.3 | 100.0 | 91.7 | 91.1 | 98.3 | 66.5 | 100.0 |
| 93 | 11.Mar.19 | 16.91 | 92.5 | 93.3 | 88.9 | 91.7 | 94.6 | 95.0 | 66.5 | 100.0 |
| 94 | 11.Mar.19 | 15.00 | 70.5 | 90.0 | 53.1 | 91.7 | 84.6 | 51.7 | 66.5 | 87.5 |
| 95 | 11.Mar.19 | 15.69 | 45.0 | 37.8 | 44.4 | 33.3 | 55.8 | 43.3 | 16.6 | 62.5 |
| 96 | 11.Mar.19 | 17.63 | 81.4 | 62.2 | 94.4 | 91.7 | 83.9 | 78.3 | 66.5 | 100.0 |
| 97 | 12.Mar.19 | 17.15 | 80.5 | 78.9 | 86.1 | 91.7 | 76.8 | 80.0 | 66.5 | 87.5 |
| 98 | 12.Mar.19 | 14.88 | 43.7 | 44.4 | 30.6 | 58.3 | 36.5 | 55.0 | 33.3 | 50.0 |
| 99 | 12.Mar.19 | 17.14 | 93.0 | 93.3 | 97.2 | 100.0 | 89.3 | 95.0 | 66.5 | 100.0 |
| 100 | 12.Mar.19 | 15.69 | 84.0 | 85.5 | 86.1 | 87.5 | 83.9 | 85.0 | 66.5 | 75.0 |
